# New and Recurring Food Insecurity During and After the COVID-19 Pandemic

**DOI:** 10.1001/jamahealthforum.2025.3603

**Published:** 2025-09-05

**Authors:** Elise Sheinberg, Noura E. Insolera, Nour M. Hammad, Alessandra Uriarte, Christine M. Weston, Melissa N. Laska, Julia A. Wolfson, Cindy W. Leung

**Affiliations:** 1 Department of Nutrition, Harvard T.H. Chan School of Public Health, Boston, Massachusetts; 2 Institute of Social Research, University of Michigan, Ann Arbor; 3 Department of Health Management and Policy, Johns Hopkins Bloomberg School of Public Health, Baltimore, Maryland; 4 Division of Epidemiology and Community Health, School of Public Health, University of Minnesota, Minneapolis; 5 Department of International Health, Johns Hopkins University Bloomberg School of Public Health, Baltimore, Maryland

## Abstract

This survey study examines the prevalence of new and recurring household food insecurity among US households during and after the COVID-19 pandemic.

## Introduction

Food insecurity (FI) was of great concern at the start of the COVID-19 pandemic. Despite research showing high FI in early 2020, national estimates of FI in 2020 (10.5%) and 2021 (10.2%) remained unchanged from 2019 (10.5%); however, FI estimates increased to 12.8% in 2022 and 13.5% in 2023.^1^ The expiration of economic supports and reductions in federal nutrition program benefits likely contributed to these increases.^2^ Longitudinal data are required to understand the proportion of US households experiencing new or recurring FI during this period. We assessed household FI dynamics from 2019 to 2023 using a nationally representative sample.

## Methods

We analyzed households from all US states and Washington, DC, that completed the 2019, 2021, and 2023 Panel Study of Income Dynamics surveys with complete information on FI and Supplemental Nutrition Assistance Program (SNAP) participation. The University of Michigan Institutional Review Board approved the study; participants provided written informed consent.

FI was assessed biennially with the US Department of Agriculture 18-item Household Food Security Survey Module. Households with low or very low FI were categorized as having FI; FI was further classified as new vs recurring relative to the previous wave. Sociodemographic characteristics included race and ethnicity (Black, Hispanic, and White), household income, SNAP participation, and children present in the household. Race and ethnicity data were obtained from survey responses and were collected because of known disparities in FI.

Statistical analyses were performed from June to November 2024 using R, version 2023.06.1 + 524 (R Project for Statistical Computing). Household-level longitudinal weights were applied to make nationally representative estimates. We evaluated differences in FI dynamics by race and ethnicity, household income, SNAP participation, and presence of children using Rao-Scott χ^2^ tests. Two-sided statistical significance was set at *P* < .05.

## Results

**Figure 1.**
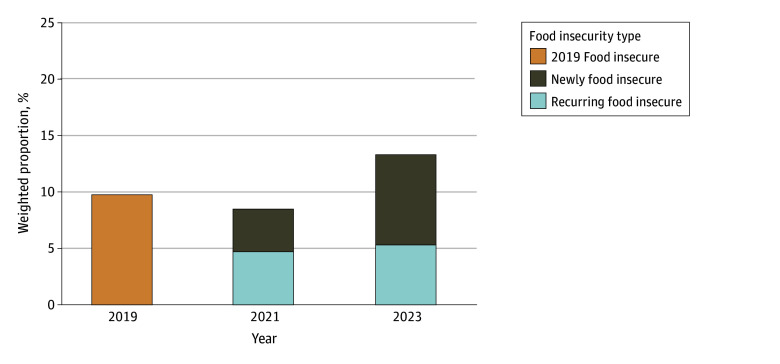
Overall Trends in Food Insecurity Among US Households by Year

Weighted FI prevalence for the full sample of 6676 households was 9.7% (720 households) in 2019, 8.5% (720 households) in 2021, and 13.3% (1163 households) in 2023 (Figure 1). Of these households, 3610 (16.7%) were Black, 709 (13.0%) were Hispanic, and 3357 (70.3%) were White. Recurring FI rates increased from 4.7% (380 households) in 2021 to 5.3% (457 households) in 2023. New FI rates increased from 3.8% (340 households) in 2021 to 8.0% (340 households) in 2023. Of households with FI, 44.5% (340 households) were new and 55.5% (380 households) were recurring in 2021; by 2023, 60.2% (706 households) were new and 39.8% (457 households) were recurring.

**Figure 2.**
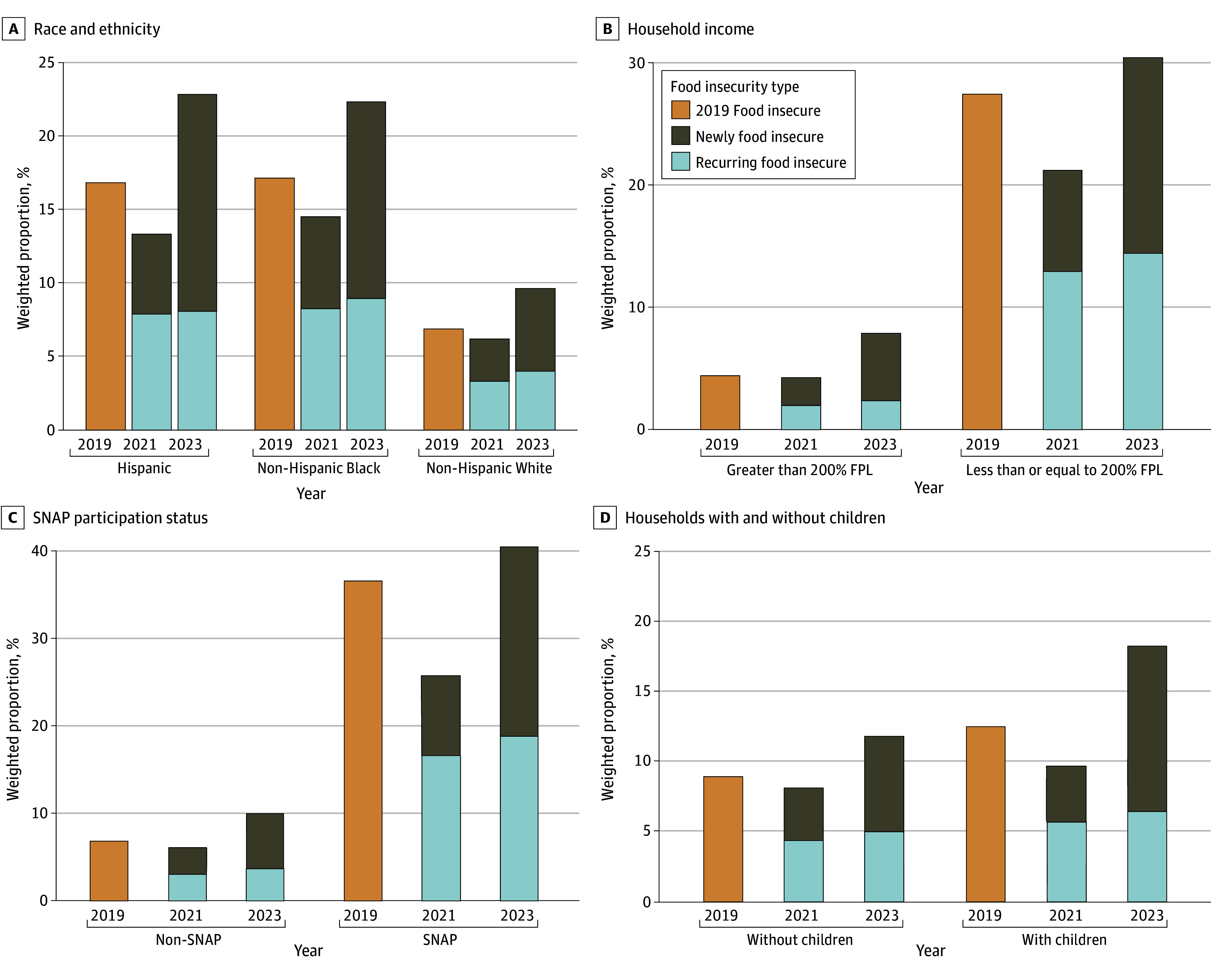
Trends in Food Insecurity Among US Households by Year, Stratified by Race and Ethnicity, Household Income, Supplemental Nutrition Assistance Program (SNAP) Participation Status, and Households With and Without Children FPL indicates federal poverty level.

FI decreased from 2019 to 2021 by a greater magnitude for socially disadvantaged groups but then increased in 2023 (Figure 2). From 2021 to 2023, new FI increased by 7.1 percentage points (pp) (95% CI, 6.4-7.8 pp) among Black households, 9.3 pp (95% CI, 8.5-10.1 pp) among Hispanic households, and 2.7 pp (95% CI, 2.5-2.9 pp) among White households. FI rates decreased by 6.3 pp (95% CI, 5.4-7.1 pp) among low-income households from 2019 to 2021 but increased by 9.1 pp (95% CI, 8.2-9.9 pp) from 2021 to 2023. New FI among low-income households increased from 7.6 pp (95% CI, 7.1-8.2 pp) from 2021 to 2023. For SNAP participants, FI decreased by 11.1 pp (95% CI, 9.5-12.2 pp) from 2019 to 2021; new FI increased by 12.5 pp (95% CI, 11.5-13.5 pp) between 2021 and 2023. Among households with children, FI decreased by 2.8 pp (95% CI, –2.2 to –3.4 pp) but increased by 8.5 pp (95% CI, 7.9-9.2 pp) in 2023.

## Discussion

In this survey study examining FI in the US during and after the COVID-19 pandemic, FI declined among all subgroups between 2019 and 2021 but exceeded prepandemic levels by 2023; new FI accounted for much of that increase. The increase in new FI is concerning, as these households face elevated risk for adverse health outcomes.^3,4,5,6^

Study limitations include the biennial FI measurement and possible recall bias. The observed trends coincide with expansions and subsequent reductions in social supports. Sustained policies are needed to meet national goals of cutting FI rates in half.

## Notes

Supplement.
Data Sharing Statement

## References

[1] Rabbitt MP, Reed-Jones M, Hales LJ, Burke MP. Household Food Security in the United States in 2023. US Dept of Agriculture, Economic Research Service; 2024. doi:10.32747/2024.8583175.ers

[2] Wells W, Jackson K, Leung CW, Hamad R. Food Insufficiency increased after the expiration of COVID-19 emergency allotments for SNAP benefits in 2023. Health Aff (Millwood). 2024;43(10):1464-1474. doi:10.1377/hlthaff.2023.01566 39374457 PMC11584048

[3] Gundersen C, Ziliak JP. Food insecurity and health outcomes. Health Aff (Millwood). 2015;34(11):1830-1839. doi:10.1377/hlthaff.2015.0645 26526240

[4] Leung CW, Epel ES, Ritchie LD, Crawford PB, Laraia BA. Food insecurity is inversely associated with diet quality of lower-income adults. J Acad Nutr Diet. 2014;114(12):1943-53.e2. doi:10.1016/j.jand.2014.06.353 25091796

[5] Bruening M, Dinour LM, Chavez JBR. Food insecurity and emotional health in the USA: a systematic narrative review of longitudinal research. Public Health Nutr. 2017;20(17):3200-3208. doi:10.1017/S1368980017002221 28903785 PMC10261670

[6] Thomas MMC, Miller DP, Morrissey TW. Food insecurity and child health. Pediatrics. 2019;144(4):e20190397. doi:10.1542/peds.2019-0397 31501236

