# Supplementary material for: New and Recurring Food Insecurity During and After the COVID-19 Pandemic
Source: JAMA Health Forum. 2025 Sep 5;6(9):e253603. doi: 10.1001/jamahealthforum.2025.3603 (PMC12413649; doi:10.1001/jamahealthforum.2025.3603)
Supplement: Supplement. — Data Sharing Statement [file jamahealthforum-e253603-s001.pdf]

Data Sharing Statement

Sheinberg. New and Recurring Food Insecurity During and After the COVID-19 Pandemic. *JAMA Health Forum*. Published September 05, 2025. doi:10.1001/jamahealthforum.2025.3603

Data
Data available: Yes
Data types: Data described in the manuscript and code book
How to access data: Data are publicly available at https://simba.isr.umich.edu/data/data.aspx
When available: With publication

Supporting Documents
Document types: None

Additional Information
Who can access the data: Anyone
Types of analyses: Any purpose
Mechanisms of data availability: Analytic code will be made available upon request pending application and approval
